# Acute Kidney Injury and Neurodevelopmental Outcomes in Extremely Premature Neonates: A Secondary Analysis of a Randomized Clinical Trial

**DOI:** 10.1001/jamanetworkopen.2025.43270

**Published:** 2025-11-12

**Authors:** Mina Hanna, Valerie Y. Chock, Nivedita Kamath, Anjali Raj, Jonathan R. Swanson, Russell Griffin, David J. Askenazi, Saudamini Nesargi

**Affiliations:** 1 Department of Pediatrics, University of Kentucky, Lexington; 2 Department of Pediatrics, Stanford University School of Medicine, Palo Alto, California; 3 St John’s Medical College Hospital, Bengaluru, Karnataka, India; 4 Department of Pediatrics, University of Virginia School of Medicine, Charlottesville; 5 Department of Pediatrics, University of Alabama at Birmingham

## Abstract

**Importance:**

Extremely low gestational age neonates are at risk of neurodevelopmental impairment (NDI). Limited data exist on the association between acute kidney injury (AKI) and NDI in this population.

**Objective:**

To describe neurodevelopmental outcomes in a cohort of extremely low gestational age neonates with AKI.

**Design, Setting, and Participants:**

This study is a secondary analysis of the Preterm Erythropoietin Neuroprotection Trial (PENUT), a phase 3 placebo-controlled randomized clinical trial of erythropoietin conducted from December 2013 to September 2016 in 30 neonatal intensive care units in the US. Participants were extremely premature neonates born at 24 to 27 weeks’ gestation. Data were analyzed in February 2024.

**Exposure:**

AKI was the primary exposure. Key aspects of AKI were evaluated in this secondary analysis, including the stage of AKI, and timing (early defined as within the first 7 days after birth; late defined as after the first 7 days).

**Main Outcomes and Measures:**

The primary outcome for this secondary analysis was the composite outcome of death or moderate to severe NDI (moderate to severe cerebral palsy, Bayley Scales of Infant Development Third Edition [BSID-III] cognitive score <85, or BSID-III motor score <85) at 22 to 26 months’ corrected gestational age. The secondary outcomes were the individual components of the composite outcome.

**Results:**

Of 941 enrolled neonates in PENUT, 660 had a follow-up visit with available BSID-III performed per protocol during the follow-up window of 22 to 26 months’ corrected gestational age. Among the survivors, 256 neonates (39%) had AKI. Neonates with AKI had significantly lower mean (SD) GA (25.1 [1.1] weeks vs 25.7 [1.1] weeks), lower birth weight (755.3 [180.9] g vs 824.8 [189.4] g), were more likely to have an Apgar score of less than 5 at 5 minutes (24.1% vs 17.1%), and 57% were male. Those with AKI had higher odds of the composite outcome (adjusted odds ratio [AOR], 1.53; 95% CI, 1.07-2.18) and higher odds of cognitive BSID-III scores less than 85 (AOR, 1.94; 1.25-2.99). Neither the stage nor timing of AKI had an association with NDI.

**Conclusions and Relevance:**

In this secondary analysis of a randomized clinical trial, AKI in preterm infants was associated with poor neurodevelopmental outcomes at 22 to 26 months’ corrected gestational age that was assessed by BSID-III scores.

**Trial Registration:**

ClinicalTrials.gov Identifier: NCT01378273

## Key Points

**Question:**

Is acute kidney injury (AKI) in extremely premature infants associated with poor neurodevelopmental outcomes?

**Findings:**

In this secondary analysis of a randomized clinical trial of 660 extremely low gestational age neonates, acute kidney injury in the first 2 weeks after birth was independently associated with death and/or severe neurodevelopmental impairment at 2 years of age after controlling for potential confounders.

**Meaning:**

Results of this study suggest that extremely low gestation in neonates who developed AKI was associated with worse performance on cognitive developmental scores, even when controlling for potential confounders.

## Teaser

This secondary analysis of a randomized clinical trial assesses the association of acute kidney injury among extremely premature neonates with neurodevelopmental outcomes at 22 to 26 months.

## Introduction

In recent decades several advances in neonatal medicine have enhanced survival rates for preterm infants, but in most cases the incidence of major morbidities has changed very little, particularly for extremely premature infants.^1^ Neurodevelopmental impairment (NDI) is currently a key outcome measure that broadly captures a wide spectrum of cognitive, motor, vision, and hearing impairment.^2^ Acute kidney injury (AKI) in the first 2 weeks of life may be a modifiable risk factor for long-term NDI; yet studies on the relationship between AKI and neurodevelopment are lacking. Understanding the early pathogenesis of preterm infants’ morbidities, with a focus on prevention and mitigation strategies, is crucial for improving their neurodevelopmental outcomes .

In animal models of AKI, neurotransmitters have recently been demonstrated to play a role in uremic adult male rats. Decreased dopamine turnover in the striatum, mesencephalon, and hypothalamus were noted 48 hours after bilateral kidney ischemia reperfusion.^3^ Clinical studies demonstrate earlier onset of multiorgan failure and increased mortality after AKI that cannot be explained by increased severity of comorbid conditions alone.^4^ A recent study showed an association between AKI in the first 2 weeks after birth with brain insult in very preterm neonates, especially in the cerebellum as assessed by MRI at corrected term age.^5^ The impact of neonatal AKI on the neurodevelopmental outcomes of preterm infants is unknown.

The Preterm Erythropoietin Neuroprotection Trial (PENUT) was a randomized clinical trial designed to test the safety and efficacy of erythropoietin as a neuroprotective agent in extremely low gestational age neonates.^6^ It was powered for the primary outcome of death or NDI assessed by Bayley Scales of Infant Development Third Edition (BSID-III) at 2 years of age. The aim of this current secondary study was to investigate the association between neonatal AKI in extremely low gestational age neonates enrolled in the PENUT trial and their neurodevelopmental outcome at 22 to 26 months’ corrected gestational age. We hypothesized that extremely preterm infants with AKI would have lower neurodevelopmental scores as assessed by BSID-III.

## Methods

### Study Design and Population

PENUT was a phase 3 placebo-controlled randomized clinical trial of erythropoietin neuroprotection in infants born at gestational ages of 24 weeks (0/7 days) to 27 weeks (6/7 days). A total of 941 infants were enrolled at 19 sites consisting of 30 neonatal intensive care units across the US from December 1, 2013, to September 31, 2016, 936 (99.5%) of whom received the first study drug dose and were considered for further analysis. This secondary analysis was performed in February 2024 and followed the Strengthening the Reporting of Observational Studies in Epidemiology (STROBE) reporting guideline for observational studies. The institutional review board at the University of Washington served as the central institutional review board, and each center involved in the study received approval from their institutional review boards or human research ethics committees. Informed written consent was obtained from the parent or legal guardian. Patients were excluded if they had known major life-threatening anomalies, known or suspected chromosomal anomalies, disseminated intravascular coagulopathy, polycythemia, hydrops fetalis, or a known congenital infection. This secondary analysis population included all infants enrolled in PENUT who survived and completed BSID-III assessments at 24-month follow-up. The trial protocol for the PENUT study is available in Supplement 1.

### Primary Exposure and Outcomes

AKI was defined by the modified KDIGO (Kidney Disease Improving Global Outcomes) criteria in the same manner that has been used to report the prevalence, biomarkers, and outcomes in this cohort.^7^ This definition has been widely used across multiple studies to evaluate neonatal AKI based on serum creatinine and urine output.^8^ Key aspects of AKI were evaluated in this secondary analysis, including the stage of AKI and timing (early defined as within the first 7 days after birth; late defined as after the first 7 days).

The primary outcome of interest was the same primary outcome for the PENUT trial, death or moderate to severe neurodevelopmental impairment at 22 to 26 months of corrected gestational age based on BSID-III, which has 3 composite components: cognitive, motor, and language scores as previously reported for this cohort.^6^ Severe NDI was defined as the presence of any of the following: severe cerebral palsy (CP), (defined as Gross Motor Function Classification System [GMFCS] >2), BSID-III cognitive score less than 70, or BSID-III motor score less than 70; and moderate to severe NDI, defined as the presence of any of the following: moderate to severe CP (GMFCS ≥2), BSID-III cognitive score less than 85, or BSID-III motor score less than 85.

### Statistical Analysis

All statistical inferences used generalized estimating equations, robust SEs, and an exchangeable working correlation structure clustered by study site as fixed effect. A generalized estimating equation logistic regression model (clustered by study site) was used to estimate odds ratios (ORs) and 95% CIs for the association between AKI and the composite outcome of death or moderate or severe NDI at 22 to 26 months’ corrected gestational age. The selection of potential confounding variables was based on significant univariate analysis at baseline. The final adjusted model included gestational age, maternal age, delivery method, delayed cord clamping, neonatal sex, birth weight, and 5-minute Apgar scores. Statistical significance was set at *P* < .05 for each key outcome. SAS version 9.4 (SAS Institute) was used for all analyses.

## Results

Of the 941 study participants enrolled in the original study, 660 (70%) survived and had 2-year neurodevelopmental outcomes performed per protocol as part of the PENUT Trial and were included in the study population for this secondary analysis. A total of 281 infants were excluded from the current secondary data analysis: not randomized (n = 5), died prior to discharge from the neonatal intensive care unit (n = 94), died prior to 22 to 26 months’ corrected gestational age (n = 12), and lost to follow-up (n = 170). The Figure shows the flow of flow infants enrolled, randomized to erythropoietin or placebo, and neurodevelopmental outcomes analyzed.

**Figure.**
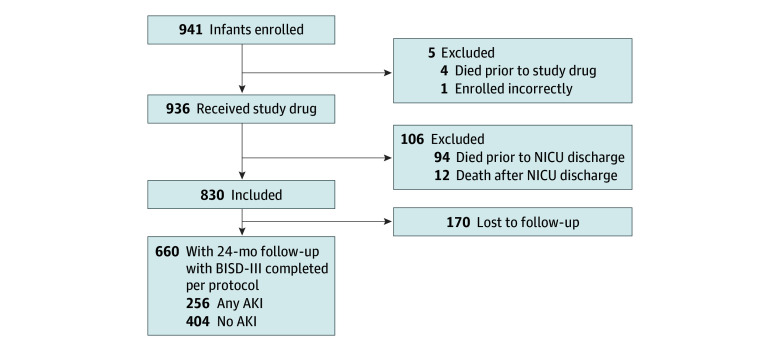
Flow Diagram of Secondary Analysis AKI indicates acute kidney injury; BSID-III, Bayley Scales of Infant Development Third Edition; NICU, neonatal intensive care unit.

Maternal demographics and perinatal and neonatal characteristics of those who survived are shown in Table 1, which depicts differences between the 256 neonates (39%) with AKI and 404 (61%) without AKI. Neonates who developed AKI had significantly lower mean (SD) gestational age (25.2 [1.1] weeks vs 25.8 [1.0] weeks), lower mean (SD) birth weight (771.5 [181.0] g vs 838.1 [85.5] g), were more likely to have an Apgar score of less than 5 at 5 minutes (21.9% vs 14.5%), were more likely to be born by Cesarean delivery, and were more often male (57%). Neonates who had delayed cord clamping were also more likely to develop AKI (41.6% vs 34.4%).

**Table 1.** Maternal Demographics and Neonatal Data

Characteristic	Participants, No. (%)		*P* value^a^
	No AKI (n = 404)	AKI (n = 256)	
Maternal			
Age, mean (SD), y	29.6 (5.9)	28.1 (6.3)	.002
Race^b^			
Black	85 (21.0)	64 (25.0)	
White	278 (68.8)	172 (67.2)	.35
Other, unknown, or unreported	41 (10.1)	20 (7.8)	
Educational level			
High school or less	122 (32.7)	85 (38.6)	.26
Some college	131 (35.1)	76 (34.5)	
College degree or greater	120 (32.2)	59 (26.8)	
Chorioamnionitis	53 (13.1)	35 (13.7)	.84
Pregnancy-induced hypertension	28 (6.9)	19 (7.4)	.81
Prenatal steroids	372 (92.1)	234 (91.4)	.44
Prenatal magnesium sulfate	333 (82.4)	204 (80.0)	.72
Perinatal characteristics			
Delivery complications	68 (16.8)	40 (15.6)	.68
Delivery type			
Cesarean delivery (scheduled)	26 (6.4)	27 (10.5)	.009
Cesarean delivery (unscheduled)	229 (56.7)	161 (62.9)	
Vaginal birth	149 (36.9)	68 (26.6)	
Cord clamping			
Immediate	138 (34.2)	99 (38.8)	.004
Delayed	139 (34.4)	106 (41.6)	
Unknown	127 (31.4)	50 (19.6)	
Multiple gestation	107 (26.5)	64 (25.0)	.67
Erythropoietin administration	201 (49.8)	120 (46.9)	.47
Neonatal			
Male sex	192 (47.5)	146 (57.0)	.02
Birth gestational age, wk			
24	56 (13.9)	97 (37.9)	<.001
25	95 (23.5)	71 (27.7)	
26	121 (30.0)	38 (14.8)	
27	132 (32.7)	50 (19.5)	
Mean (SD)	25.8 (1.0)	25.2 (1.1)	<.001
Birth weight, mean (SD), g	838.1 (185.5)	771.5 (181.0)	<.001
Small size for gestational age	52 (12.9)	38 (14.9)	.47
Apgar score at 1 min, median (IQR)	4 (2-6)	4 (2-6)	.03
Apgar score at 5 min <5	58 (14.5)	56 (21.9)	.014
IVH	38 (9.4)	35 (13.7)	.09
Dexamethasone administration >14 d	19 (4.7)	22 (8.6)	.04

Abbreviations: AKI, acute kidney failure; IVH, intraventricular hemorrhage.

^a^ Based on χ^2^ for categorical variables and *t* test for continuous variables.

^b^ Race was reported by the mother. No further breakdown is available.

Results of the primary analysis for the associations between AKI and BSID-III scores (cognitive, motor, and language components) are shown in Table 2. The primary outcome of death or moderate to severe NDI was higher in infants who had any AKI (55.4% vs 39.4%; *P* = .02). Accounting for potential confounding variables, neonates with AKI had 1.56 (95% CI, 1.05-2.34) higher adjusted odds of death or severe NDI and 1.53 (95% CI, 1.07-2.18) higher adjusted odds of death or moderate to severe NDI.

**Table 2.** Differences in Neurodevelopmental Outcomes Between Those With and Without Any AKI

Outcome	Participants, No./total No. (%)		AOR (95% CI)^a^	*P* value
	Without any AKI (n = 453)	With any AKI (n = 307)		
Death	49/453 (10.8)	51/307 (16.6)	1.26 (0.85-1.87)	.26
Death or severe NDI	81/435 (17.9)	95/307 (30.9)	1.56 (1.05-2.34)	.03
Severe CP	5/400 (1.3)	6/256 (2.3)	1.98 (0.51-7.64)	.32
BSID-III cognitive <70	17/404 (4.2)	31/256 (12.1)	2.45 (1.47-4.09)	.001
BSID-III motor <70	27/397 (6.8)	38/252 (15.1)	1.64 (0.72-3.73)	.24
Death or moderate or severe NDI	177/453 (39.1)	170/307 (55.4)	1.53 (1.07-2.18)	.02
Moderate or severe CP	9/400 (2.3)	16/256 (6.3)	3.05 (1.33-7.02)	.01
BSID-III cognitive <85	81/404 (20.0)	95/256 (37.1)	1.94 (1.25-2.99)	.003
BSID-III motor <85	105/397 (26.4)	91/252 (36.1)	1.22 (0.82-1.82)	.33

Abbreviations: AKI, acute kidney injury; AOR, adjusted odds ratio; CP, cerebral palsy; BSID-III, Bayley Scales of Infant Development Third Edition; NDI, neurodevelopmental impairment.

^a^ Based on general estimating equation logistic regression (clustered by study site) and adjusting for maternal age, delivery method, delayed cord clamping, neonatal sex, gestational age, birth weight, and 5-minute Apgar score.

Moreover, when focusing on the BSID-III cognitive component, neonates with AKI had a 1.94 (95% CI, 1.25-2.99) higher adjusted odds of cognitive scores less than 85 compared with those without AKI. Adjusting for interaction of sex with AKI status, the findings were not statistically significant (eTable in Supplement 2). The best-case/worst case sensitivity analysis to explore whether those who did not return for follow-up could have altered the conclusions did not find any significant difference that affected the overall findings. Adding erythropoietin and/or dexamethasone administration to the model did not change the findings for cofactors and NDI.

There were no significant differences between the severity of AKI and any neurodevelopmental impairment (Table 3). However, cognitive delay was associated with mild AKI (stage 1) and severe AKI (stage 2 or 3). The timing of AKI had no association with NDI (Table 4).

**Table 3.** Association Between Stage of AKI and Any NDI

AKI stage	AOR (95% CI)^a^			
	Any delay	Cognitive delay	Motor delay	Language delay
No AKI	1 [Reference]	1 [Reference]	1 [Reference]	1 [Reference]
Mild (stage 1)	1.45 (0.95-2.20)	2.18 (1.38-3.45)	1.23 (0.73-2.08)	1.51 (0.94-2.42)
Severe (stage 2 or 3)	0.93 (0.65-1.32)	1.74 (1.07-2.83)	1.21 (0.85-1.74)	0.97 (0.64-1.46)

Abbreviations: AKI, acute kidney injury; AOR, adjusted odds ratio; NDI, neurodevelopmental impairment.

^a^ Based on general estimating equation logistic regression (clustered by study site) and adjusting for maternal age, delivery method, delayed cord clamping, neonatal sex, gestational age, birth weight, and 5-minute Apgar score.

**Table 4.** Association Between Timing of AKI and Any NDI

AKI time	AOR (95% CI)^a^			
	Any delay	Cognitive delay	Motor delay	Language delay
No AKI	1 [Reference]	1 [Reference]	1 [Reference]	1 [Reference]
Early (<7 d)	1.02 (0.67-1.55)	2.14 (1.24-3.68)	0.83 (0.47-1.46)	1.02 (0.60-1.75)
Late (>7 d)	1.19 (0.85-1.69)	1.87 (1.14-3.07)	1.40 (0.93-2.10)	1.27 (0.87-1.86)

Abbreviations: AKI, acute kidney injury; AOR, adjusted odds ratio; NDI, neurodevelopmental impairment.

^a^ Based on general estimating equation logistic regression (clustered by study site) and adjusting for maternal age, delivery method, delayed cord clamping, neonatal sex, gestational age, birth weight, and 5-minute Apgar score.

## Discussion

In this secondary analysis of a large phase 3 placebo-controlled randomized clinical trial, the incidence of AKI in extremely preterm neonates was 39%, similar to other published studies.^8,9^ To our knowledge, this is one of the few studies to examine the association between AKI and neurodevelopmental outcomes in this vulnerable population. Preterm infants with AKI had a worse adjusted composite outcome of death or moderate to severe NDI and higher rates of a cognitive score of less than 70 on the BSID-III, and moderate to severe cerebral palsy. Mild and severe AKI were associated with cognitive impairment. Similarly, both early and late AKI were associated only with the cognitive developmental domain in this cohort. The present study did not show a statistically significant increase in mortality after adjusting for confounders, similar to a retrospective single-center cohort study of very low-birth-weight infants.^10^ However, other large epidemiological studies have shown an increased risk of mortality in preterm infants with AKI, especially in extremely low birth weight neonates.^8,11,12^

Limited studies have investigated associations between AKI and neurodevelopmental outcomes. In a single-center cohort, Chen et al.^13^ assessed 732 neonates less than 31 weeks of gestational age and found that those with AKI exhibited higher rates of comorbidities and risk factors for poor outcomes included lower Apgar scores, lower gestational age, and maternal preeclampsia. Long-term follow-up revealed decreased head circumference *z* scores, higher prevalence of microcephaly, and worse neurodevelopmental outcomes in AKI survivors vs controls in unadjusted analysis. Nevertheless, some findings were significant only with the oliguric AKI group when adjusted for gestational age, sex, and other covariates.^13^ Al-Mouqdad et al^5^ showed that very low birth weight neonates with AKI in the first 2 weeks after birth had abnormal MRI findings at term corrected age: cerebellum signal abnormalities, cerebellar volume reduction, and a high total cerebellum score. While neurodevelopmental outcomes were not assessed, abnormal MRI findings at term corrected age are known to correlate with abnormal neurodevelopment later in childhood.^14^ A retrospective study of preterm infants born before 33 weeks of gestational age found that creatinine values greater than 1.6 mg/dL at 24 to 27 weeks of gestation, 1.1 mg/dL at 28 to 29 weeks of gestation, and 1.0 mg/dL at 30 to 32 weeks of gestation (to convert creatinine to micromoles per liter, multiply by 88.4) were associated with mortality before and after adjustment for risk factors, and with nonoptimal neurodevelopmental outcome, before adjustment.^15^ In the term neonatal population, a recent study of 203 infants who underwent cardiac surgery, showed that those with recurrent episodes of AKI had a delay in one or more domains of the Bayley scale and had significantly lower scores in all 3 domains, namely, cognitive, language, and motor.^16^

The mechanisms by which AKI may affect neurodevelopmental outcomes are unclear and this study is not equipped to identify a causal relationship. A plausible explanation for adverse neurodevelopment could be based on the kidney-brain cross talk in the setting of AKI in preterm infants. Growing evidence in animal models suggests that AKI is not an isolated event but results in remote organ dysfunction involving the heart, lungs, and brain through an inflammatory mechanism that involves neutrophil migration, cytokine expression, and increased oxidative stress.^4^ In mice, kidney ischemia reperfusion resulted in increased levels of pro-inflammatory chemokines, which increased expression of glial fibrillary acidic protein in astrocytes in the cortex and corpus callosum.^17^ Chronic kidney dysfunction might impair growth and influence brain function in the pediatric population.^18,19^

### Strengths and Limitations

This study has several strengths. The PENUT trial included a well-characterized cohort of extremely preterm neonates with extensive standardized neurodevelopmental follow-up data. The dataset had a robust number of serum creatinine values per patient and the most contemporary definition of AKI was used, which allowed the evaluation of incidence, timing, and severity of AKI and associated neurodevelopmental outcomes.

The findings should be interpreted with caution as there are some inherent limitations to the study design. This is a secondary analysis; therefore, findings are limited to associations as residual confounding factors may remain in this analysis. We also note that other variables, such as severity of illness, nutrition, and different clinical practices, may result in changes in neurodevelopmental outcomes. Neurodevelopmental testing at 2 years of age may provide less reliable information than assessments performed at older ages. Hearing and vision assessments were not included. A multicenter study with long-term prospective follow-up is warranted to delineate the kidney consequences after AKI in very preterm neonates.

## Conclusions

In this multicenter, retrospective secondary analysis of a randomized clinical trial, we found that AKI in preterm infants was associated with poor neurodevelopmental outcomes measured at 2 years of age. Given the long-term health outcomes, it is essential that clinicians accurately diagnose and track AKI in preterm infants. Whether the findings may be causal or whether AKI is a factor associated with poor neurodevelopmental outcome or both needs further exploration.

## Notes

Supplement 1. The PENUT Trial Protocol

Supplement 2. **eTable.** Adjusted models with an interaction of sex with AKI status

Supplement 3.
Data Sharing Statement
