# Supplementary material for: Acute Kidney Injury and Neurodevelopmental Outcomes in Extremely Premature Neonates: A Secondary Analysis of a Randomized Clinical Trial
Source: JAMA Netw Open. 2025 Nov 12;8(11):e2543270. doi: 10.1001/jamanetworkopen.2025.43270 (PMC12612947; doi:10.1001/jamanetworkopen.2025.43270)
Supplement: Supplement 2. — eTable. Adjusted models with an interaction of sex with AKI status [file jamanetwopen-e2543270-s002.pdf]

Supplemental Online Content

Hanna M, Chock VY, Kamath N, et al. Acute kidney injury and neurodevelopmental outcomes in extremely premature neonates: a secondary analysis of a randomized clinical trial. *JAMA Netw Open.* 2025;8(11):e2543270. doi:10.1001/jamanetworkopen.2025.43270

eTable. Adjusted models with an interaction of sex with AKI status

This supplemental material has been provided by the authors to give readers additional information about their work.

© 2025 Hanna M et al.]. *JAMA Network Open*.

	Males	Females	*p*-value
Death	1.08 (0.57-2.06)	1.51 (0.93-2.44)	0.45
Death/Severe NDI	1.45 (0.85-2.47)	1.72 (0.96-3.10)	0.67
Severe CP	2.73 (0.77-9.69)	0.50 (0.04-6.86)	0.24
BSID-III cognitive <70	3.53 (1.45-8.60)	1.45 (0.52-4.05)	0.18
BSID-III motor <70	1.95 (0.94-4.05)	1.22 (0.48-3.06)	0.42
Death/Moderate/severe NDI	1.71 (1.10-2.66)	1.34 (0.84-2.15)	0.45
Moderate/severe CP	4.15 (1.30-13.32)	1.65 (0.34-8.03)	0.36
BSID-III cognitive <85	2.07 (1.25-3.44)	1.79 (1.02-3.14)	0.69
BSID-III motor <85	1.41 (0.85-2.34)	1.02 (0.58-1.78)	0.38
*Based on general estimating equation logistic regression (clustered by study site) and adjusting for maternal age, delivery method, delayed cord clamping, neonatal sex, gestational age, birthweight, and 5-minute Apgar<br>CP: cerebral palsy, BSID-III: Bayley Scales of Infant Development Third Edition			

eTable. Ajusted models with an interaction of sex with AKI status

© 2025 Hanna M et al.]. *JAMA Network Open*.
