# Supplementary material for: Acute Kidney Injury and Neurodevelopmental Outcomes in Extremely Premature Neonates: A Secondary Analysis of a Randomized Clinical Trial
Source: JAMA Netw Open. 2025 Nov 12;8(11):e2543270. doi: 10.1001/jamanetworkopen.2025.43270 (PMC12612947; doi:10.1001/jamanetworkopen.2025.43270)
Supplement: Supplement 3. — Data Sharing Statement [file jamanetwopen-e2543270-s003.pdf]

Data Sharing Statement

Hanna. Acute Kidney Injury and Neurodevelopmental Outcomes in Extremely Premature Neonates. *JAMA Netw Open*. Published November 12, 2025.
doi:10.1001/jamanetworkopen.2025.43270

Data
Data available: Yes
Data types: Deidentified participant data
How to access data: NIH
When available: With publication

Supporting Documents
Document types: Other (please specify)
Additional Information: Primary study data available through NIH
How to access documents: Through NIH
When available: With publication

Additional Information
Who can access the data: anyone requesting the data
Types of analyses: for a specified purpose
Mechanisms of data availability: after approval of a proposal
